# A multistep image analysis method to increase automated identification efficiency in immunohistochemical nuclear markers with a high background level

**DOI:** 10.1186/1746-1596-8-S1-S13

**Published:** 2013-09-30

**Authors:** Marylène Lejeune, Vanessa Gestí, Barbara Tomás, Anna Korzyńska, Albert Roso, Cristina Callau, Ramon Bosch, Jordi Baucells, Joaquín Jaén, Carlos López

**Affiliations:** 1 Molecular Biology and Research Section, Hospital de Tortosa Verge de la Cinta, IISPV, URV, Tortosa, Spain; 2 Unitat de Suport a la Recerca de la Gerencia Territorial Terres de l’Ebre, IDIAP Jordi Gol, IISPV, URV, UAB, Tortosa, Spain; 3 Department of Pathology, Hospital de Tortosa Verge de la Cinta, IISPV, Tortosa, Spain; 4 Laboratory of Processing Systems of Microscopic Image Information, Nalecz Institute of Biocybernetics and Biomedical Engineering, Polish Academy of Sciences, Warsaw, Poland; 5 Department of Informatics, Hospital de Tortosa Verge de la Cinta, IISPV, Tortosa, Spain

## Background

In anatomical and surgical pathology, the customary method of manual observation and measurement of immunohistochemically stained markers from microscopic images is tedious, expensive and time consuming. There is great demand for automated procedures for analyzing digital images (DIs) of these markers [1] given that they reduce human variability in the evaluation of stained markers [2,3] and increase the speed and efficiency of the analysis [4]. Computerized DI analysis software generally involves a stained objects/nuclei segmentation method to detect and quantify the number of positively stained markers in combination with the standard evaluation of their morphometric and/or densitometric features [5,6]. However, automatic segmentation often fails due to the presence of spurious stain deposits in tissue sections (background). The “removal” of the background from noisy DIs, so that only the objects of interest are identified, is difficult due to the color values of pixels in the nuclei and background overlapping during the color segmentation processes.

We previously developed an automated macro that allows quantification of several nuclear markers in various neoplasic tissues [7]. In an attempt to standardize the immunohistochemical analysis and to improve cell detection, we propose a new procedure that quantifies only positively stained nuclei even though they have a similar color to that of the surrounding tissue. The aim of this work was to develop a single automated procedure that allows images to be analyzed irrespective of whether the spurious stain deposit in background is present or absent. The multistep process includes algorithms that permit this discrimination so that the appropriate procedure for optimal quantification can then be applied.

## Materials and methods

### Images

Histological sections of lymphomas and breast cancer tissues, previously immunohistochemically stained with standardized protocols [8,9], were selected from the archives of the Department of Pathology of the Hospital de Tortosa Verge de la Cinta, Catalonia, Spain. Staining was performed with monoclonal antibodies directed against the nuclear protein estrogen receptors (ERs; clone NCL-ER-6F11, Novocastra, Newcastle upon Tyne, UK), progesterone receptors (PRs; NCL-PGR-312, Novocastra), Ki-67 (clone MIB-1, Dako, Carpinteria, CA, USA) and FOXP3 (clone FOXP3-236A/E7, CNIO, Spain). The entire process was standardized to ensure high reproducibility and brown staining homogeneity, which are very important requirements for image analysis [10]. This study received institutional review board approval.

### Image capture

**Figure 1:**
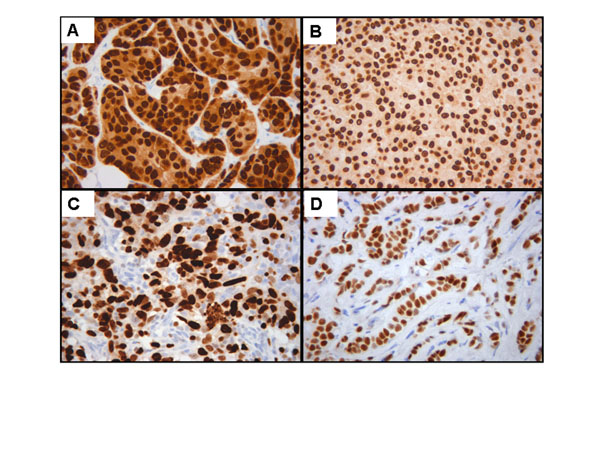
Illustration of original digital images of immunohistochemically stained nuclear markers with different background level (A, B) and without background (C, D).

Stained tissue sections were viewed using brightfield illumination under a Leica DM LB2 upright light microscope (Leica Microsystems Wetzlar GmbH, Wetzlar, Germany) with a 40x plane-apochromatic objective. One hundred digital images were captured with a Leica DFC320 digital camera connected to a computer and controlled with the Leica IM50 v4.0 program. TIFF format DIs, with a resolution of 1392 x 1040 pixels (1.4 Mpixels) in RGB 24 true-color format, were selected on the basis of the presence or absence of the spurious stain deposits of the background, otherwise ensuring a variety of concentrations and distributions of stained nuclei (Figure 1).

### Procedure developed in the new procedure

**Figure 2:**
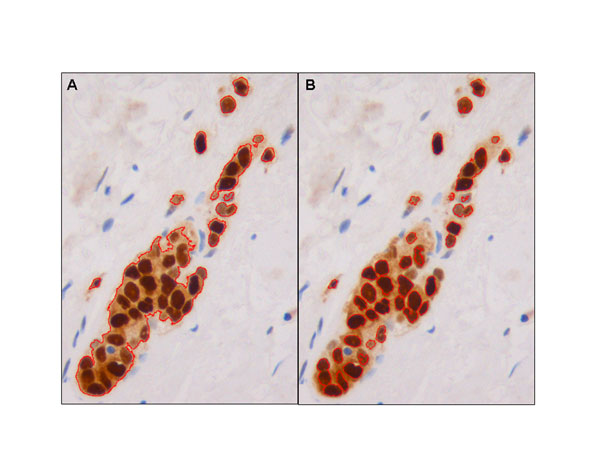
Example of active contour segmentation in digital images with positively stained nuclear markers. Evident differences in the contours obtained with the old macro (A) and those obtained with the new procedure (B).

The new automated multistep DI analysis procedure was developed with Image-Pro® Plus 5.0 software (Media Cybernetic, Silver Spring, USA). We had previously developed an automated macro to quantify stained nuclei in images without background using an RGB color model and iterative morphological segmentation [7]. This macro makes use of a wide color range to detect positive nuclei from the darkest to the lightest positive brown color pixel and applies a mask to displace the pixel color values of negative objects outside the segmentation color range of the positive nuclei. However, in DIs with a background this macro does not segment DIs correctly due to the similar color values of the positive nuclei and the background (Figure 2).

**Figure 3:**
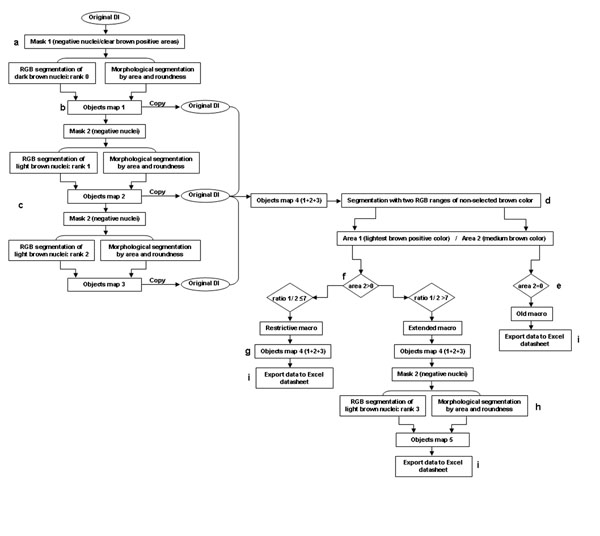
Schematic representation of the automated multistep procedure. Image analysis was carried out with the Image-Pro® Plus 5.0 program and data from selected positive objects are exported to an Excel datasheet in which appropriate algorithms are implemented, enabling the definitive number of positive cells to be calculated.

We therefore developed a multistep procedure that discriminates DIs as a function of the presence or absence of background and that enables the more appropriate of the three macros to be applied directly. First, a mask overlaps objects with negative and light positive-intensity pixels (Figure 3a) so that only positive objects with the darkest range of color (objects map 1) can be selected (Figure 3b). Then, using a mask only for negative objects, the next two steps select the clearer positive objects (objects map 2 and 3) using different color ranges and morphological ranges of area and roundness (Figure 3c). The objects map 4 is the sum of all the positive objects in the three previous steps. At the end of the positive selection, another step with a discriminative algorithm is applied in which the non-selected brown color is segmented with two different ranges of brown (Figure 3d). If no brown color is detected with the second range (area 2 = 0), the algorithm determines that there is no background in DIs that are automatically analyzed with the old macro (Figure 3e). On the other hand, when DIs with background were detected (area 2 > 0), they are submitted to another algorithm that calculate the ratio of the area of the two color ranges (Figure 3f). DIs with a low-level background (low ratio) are automatically analyzed with a restrictive macro with the first three steps of the new procedure (Figure 3g). DIs with a high background level (high ratio) are automatically analyzed with the new procedure, supplemented with a final step that selects the clearest objects using color and morphological segmentation (Figure 3h). The three macros detect only positive objects (individual nuclei and clusters). Information about the area of the positive objects detected with these macros is automatically exported to an Excel datasheet (Figure 3i) containing several algorithms so that the number of definitive positive nuclei in those selected objects can be estimated, as previously described [7].

### Quantification and statistical analysis

The mean of two manual counts made by two trained observers was taken as the reference value (gold standard) to validate the results obtained with the old macro and the newly proposed procedure. The comparisons made were: manual 1 versus manual 2 readings; mean of manual readings versus old macro reading; mean of manual readings versus new procedure reading; and the old macro versus the new procedure readings. The extents of agreement between the manual and automatic results were evaluated with Bland-Altman and Kaplan-Meier analyses with their corresponding graphs. Bland-Altman graphs illustrate the differences between the compared methods with respect to the mean of each paired count. Kaplan-Meier curves portray the conditional probability of observing differences between results obtained from the methods compared. All statistical analyses were carried out with SPSS 19.0.

## Results and discussion

**Figure 4:**
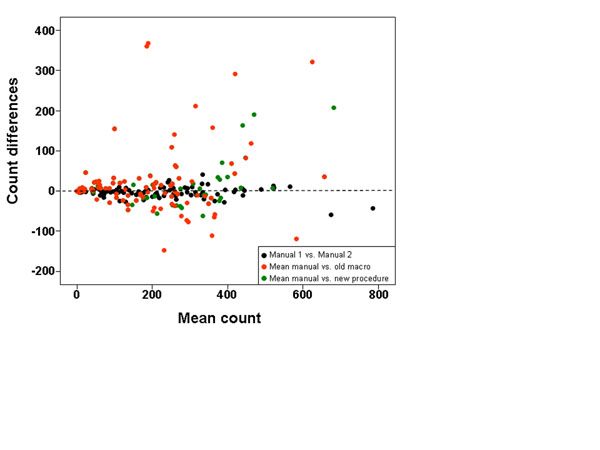
Bland-Altman graph showing differences between the two manual quantification methods (in black), the mean of the manual counts and the old macro results (in red) and the mean of the manual counts vs. the new procedure results (in green). The Y axis represents the difference between the results from pairs of methods, and the X axis represents the mean of both counts for each comparison.

**Figure 5:**
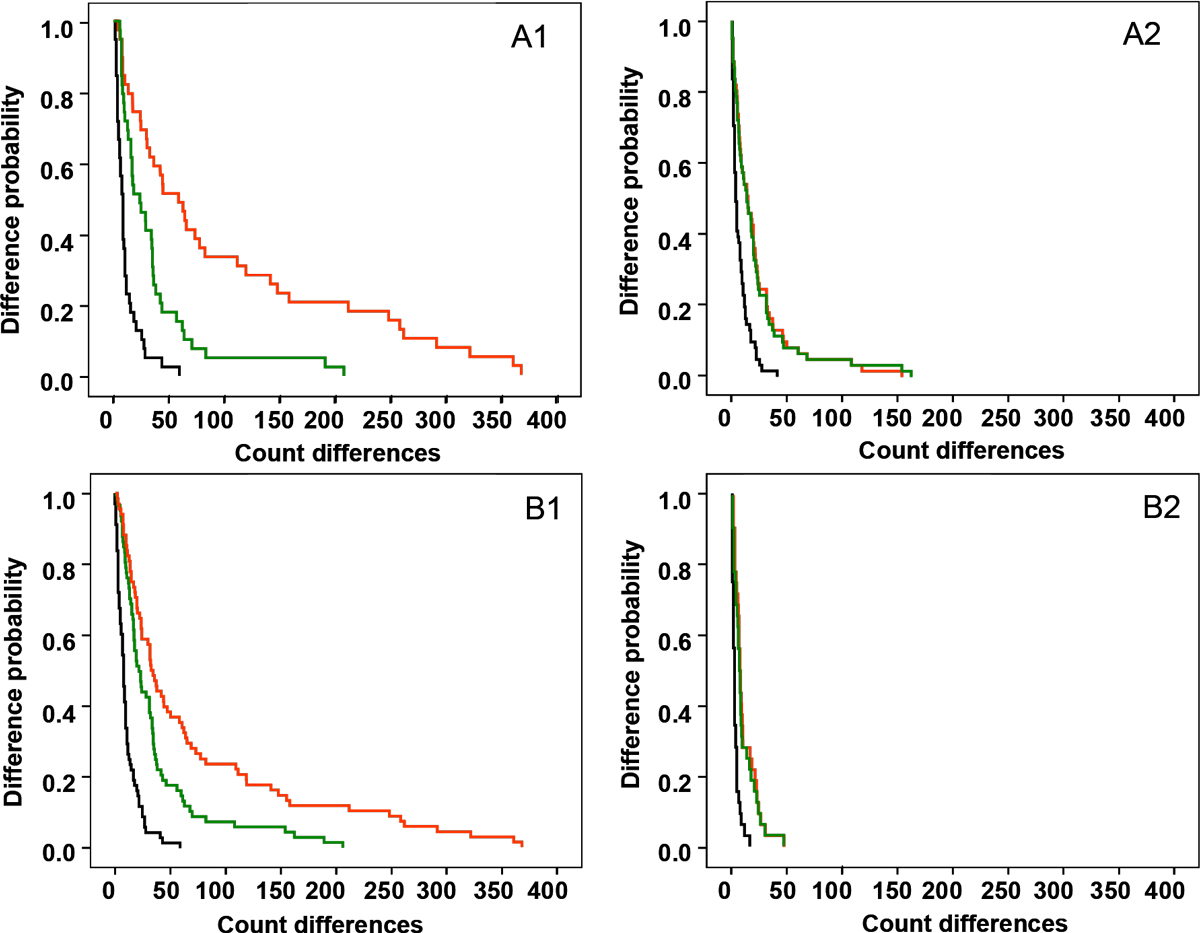
Superimposed Kaplan-Meier curves comparing the probability of difference between the manual reading 1 vs. manual reading 2 (in black), the mean of manual readings vs. old macro (in red) and mean of manual readings vs. new procedure (in green). The X axis represents count differences between the two methods or readings compared and the Y axis represents the probability of observing these differences. The upper row (A) represents the results obtained with DIs grouped in function of the background level: DIs with background (A1) versus DIs without background (A2). The lower row represents the results obtained with DIs grouped in function of their complexity: high-complexity DIs (B1) and low-complexity DIs (B2).

Overall, the Bland-Altman graph (Figure 4) indicates that count differences obtained comparing the new procedure vs. the mean of the manual counts (green circle) were lower than the old macro vs. the mean of the manual counts (red circle). Nevertheless, greater accuracy was obtained from images with background (Figure 5A1) where the probability of observing a difference of 50 nuclei between the new procedure and the manual quantification (22.7%, green curve) is less than half that between the old macro and manual quantification (47.4%; red curve).

As previously demonstrated, image complexity relative to the number of positively stained nuclei may affect the automated nuclear quantification [8]. In the present study, DIs were divided as before into a low-complexity group (≤100 positively stained nuclei/images) and a high-complexity group (>100 positively stained nuclei/image). As observed in Figure 5B2, the small differences in the counts between the manual method vs. the old macro and between manual counts vs. the new procedure were similar in low-complexity images. However, in high-complexity images (Figure 5B1), larger count differences were observed, although those between the manual counts vs. the new procedure were much lower than those between the manual counts vs. the old macro.

**Figure 6:**
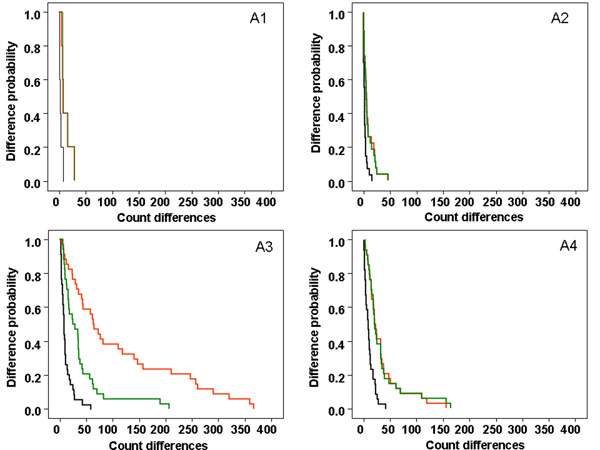
Kaplan-Meier curves comparing the probability of difference between the manual reading 1 vs. manual reading 2 (in black), the mean of manual readings vs. old macro (in red) and mean of manual readings vs. new procedure (in green). The X axis represents count differences between the two methods or readings compared and the Y axis represents the probability of observing these differences. DIs have been grouped according to their complexity and their background level: (A1) low-complexity DIs with background, (A2) low-complexity DIs without background, (A3) high-complexity DIs with background and (A4) high-complexity DIs without background.

However, when images were grouped by background and complexity (Figure 6), the differences increased more with the complexity of the DIs than with the presence of the background. High-complexity DIs with a background (Figure 6A3) gave the greatest differences between manual counts vs. old macro (red curve), with those between manual counts and the new procedure being the next biggest (green curve). High-complexity images without background (Figure 6A4) gave the same difference for the comparison between the old macro and the new procedure with the manual readings (red and green curves).

A general point regarding the proposed method is that, for high-complexity DIs with background (Figure 6A3), the quantification of nuclear markers obtained with the new procedure (green curve) was closer to the gold standard (manual method, black curve) than with the old macro (red curve). For high-complexity DIs without background (Figure 6A4), the two automated methods gave the same results (red and green curves). The presence or absence of background did not appear have a great influence on the quantification of nuclear markers in low-complexity DIs (Figure 6A1-A2), probably because these images have a lower background level than the other images.

## Conclusions

Despite specific and careful preparation of tissues and the use of blocking buffers, strong background staining can sometimes mask the detection of the target antigen during automated analysis. The results of the method presented in this paper are promising since the selective identification of brown color ranges and morphological parameters of selected objects in DIs enables the background to be discriminated during the automated localization and quantification of specific stained nuclei. The principle of this approach is applicable to all quantitative nuclear signals and should prove useful in a variety of tumor specimens, irrespective of the immunohistochemical techniques employed.

## List of abbreviations used

DIs: digital images; ERs: estrogen receptors; FOXP3: forkhead box protein 3; PRs: progesterone receptors; TIFF: Tagged Image File Format

## Competing interests

The authors declare that they have no competing interests.

## Author contributions

ML, CL and JJ conceived the study. CL designed and implemented the algorithms. JB and AK provided IT support. RB and JJ carried out the manual quantification, discussed the methods and modified them. VG, BT and CC realized the automated quantification with the two automated methods and designed the database. AR analyzed the data. ML, CL and AK drafted the manuscript. All authors revised the manuscript and approved the final version.
